# Optimal Mean Arterial Pressure for Favorable Neurological Outcomes in Survivors after Extracorporeal Cardiopulmonary Resuscitation

**DOI:** 10.3390/jcm11020290

**Published:** 2022-01-06

**Authors:** Yun Im Lee, Ryoung-Eun Ko, Jeong Hoon Yang, Yang Hyun Cho, Joonghyun Ahn, Jeong-Am Ryu

**Affiliations:** 1 Department of Internal Medicine, National Cancer Center, Goyang 10408, Korea; twirline@gmail.com; 2 Department of Critical Care Medicine, Samsung Medical Center, Sungkyunkwan University School of Medicine, Seoul 06351, Korea; ryoungeun.ko@samsung.com (R.-E.K.); jhysmc@gmail.com (J.H.Y.); 3 Division of Cardiology, Department of Medicine, Samsung Medical Center, Sungkyunkwan University School of Medicine, Seoul 06351, Korea; 4 Department of Thoracic and Cardiovascular Surgery, Samsung Medical Center, Sungkyunkwan University School of Medicine, Seoul 06351, Korea; yanghyun.cho@samsung.com; 5 Biomedical Statistics Center, Data Science Research Institute, Samsung Medical Center, Seoul 06355, Korea; jhguy.ahn@samsung.com; 6 Department of Neurosurgery, Samsung Medical Center, Sungkyunkwan University School of Medicine, Seoul 06351, Korea

**Keywords:** mean arterial pressure, extracorporeal cardiopulmonary membrane oxygenation, outcome

## Abstract

We evaluated the optimal mean arterial pressure (MAP) for favorable neurological outcomes in patients who underwent extracorporeal cardiopulmonary resuscitation (ECPR). Adult patients who underwent ECPR were included. The average MAP was obtained during 6, 12, 24, 48, 72, and 96 h after cardiac arrest, respectively. Primary outcome was neurological status upon discharge, as assessed by the Cerebral Performance Categories (CPC) scale (range from 1 to 5). Overall, patients with favorable neurological outcomes (CPC 1 or 2) tended to have a higher average MAP than those with poor neurological outcomes. Six models were established based on ensemble algorithms for machine learning, multiple logistic regression and observation times. Patients with average MAP around 75 mmHg had the least probability of poor neurologic outcomes in all the models. However, those with average MAPs below 60 mmHg had a high probability of poor neurological outcomes. In addition, based on an increase in the average MAP, the risk of poor neurological outcomes tended to increase in patients with an average MAP above 75 mmHg. In this study, average MAPs were associated with neurological outcomes in patients who underwent ECPR. Especially, maintaining the survivor’s MAP at about 75 mmHg may be important for neurological recovery after ECPR.

## 1. Introduction

A favorable neurologic outcome is one of the most important issues after cardiopulmonary resuscitation (CPR) [1]. In survivors after cardiac arrest, brain recovery depends on the prompt restoration of cerebral blood flow (CBF) to meet the metabolic demand of the brain [2]. Especially, mean arterial pressure (MAP) is one of the main factors that determine CBF [1].

However, there are limited data concerning appropriate MAP and its maintenance duration for favorable neurological outcomes after cardiac arrest [3,4,5]. American Heart Association suggested circumvention and immediate correction of MAP less than 65 mmHg in post-resuscitation care [6,7]. However, no specific target of appropriate blood pressure is known in managing post-cardiac arrest survivors [3]. Survivors after cardiac arrest may have altered cerebral autoregulation [2]. They may require much higher MAPs (above 65 mmHg) to maintain CBF and adapt to the altered autoregulation [2,8]. Especially, some studies reported much higher MAPs compared with that of the current practice guidelines for favorable neurological outcomes [9].

Furthermore, there exists no study on the evaluation of optimal blood pressure for survivors with extracorporeal cardiopulmonary resuscitation (ECPR). Neurologic outcomes may be affected by the recovery timing of native circulation, the amount of extracorporeal membrane oxygenation (ECMO) support, as well as autoregulation of CBF [1]. It is difficult to predict the effect of flow generated by ECMO on CBF autoregulation. In addition, bleeding complications such as intracerebral hemorrhage can occur due to high blood pressure and concomitant anticoagulation in patients with ECMO. Overall, the blood pressure target after ECPR may not be similar to that of conventional CPR. Therefore, we have evaluated the optimal MAP target for favorable neurologic outcomes after ECPR in this study.

## 2. Materials and Methods

### 2.1. Study Population

This was a retrospective, single-center, and observational study involving adult patients who underwent ECPR during hospitalization between January 2013 and December 2019. This study was approved by the Institutional Review Board of Samsung Medical Center (IRB No. 2019-10-119). The requirement for informed consent was waived by the Institutional Review Board of Samsung Medical Center due to the retrospective nature of the study.

**Figure 1:**
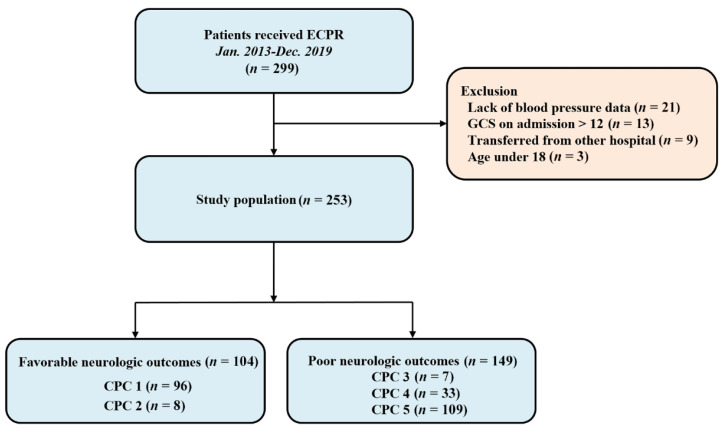
Study flow chart. ECPR, Extracorporeal Cardiopulmonary Resuscitation; GCS, Glasgow Coma Scale; CPC, Cerebral Performance Category.

All adult patients (age ≥18 years) who underwent ECPR during the study period and had a Glasgow Coma Scale (GCS) < 13 on ICU admission were considered eligible for the study. The inclusion and exclusion criteria of ECPR in the institution are as follows. (1) Inclusion: persistent cardiopulmonary arrest despite conventional CPR for 10 min; witnessed arrest; the event that caused the arrest is thought to be reversible. (2) Exclusion: unwitnessed arrest; conventional CPR undertaken for longer than 60 min at the time of initial contact for ECMO cannulation; life expectancy less than six months or limited physical activity; pre-existing severe neurologic disease or damage prior to arrest (including traumatic brain injury, major stroke or severe dementia); malignancy in terminal stage; current massive intracranial hemorrhage, arrest of traumatic origin with uncontrolled bleeding; irreversible organ failure or multiple organ failure leading to cardiac arrest; patients who previously signed “Do not resuscitate” order. Age alone did not constitute a contraindication for ECPR. When a patient with in- or out-of-hospital cardiac arrest met the inclusion criteria, ECPR was performed if needed. Furthermore, the patient was eligible for this study. We excluded patients aged under 18 years, patients who were transferred from other hospitals after ECPR, and patients with insufficient medical records. Ultimately, 253 patients were analyzed in this study (Figure 1).

### 2.2. Definitions and Outcomes

In this study, ECPR was defined as successful veno-arterial ECMO implantation and pump-on with cardiac compression during index procedure in patients with cardiac arrest [1]. When a return of spontaneous circulation occurs during ECMO cannulation, clinicians typically do not remove the cannula or stop the ECMO pump-on process [10]. The CPR duration was defined as the total time from onset to halt of chest compression. The on-site intensivists determined whether to implement the target temperature management and the temperature target according to the protocol of the hospital. Target temperature management was executed with surface cooling devices. Either a cooling blanket or a commercial temperature regulation system consisted of hydrogel pads (Arctic Sun, Medivance Corp, Louisville, CO, USA) were used.

The MAP was calculated as: (systolic arterial pressure + 2 × diastolic arterial pressure)/3. The average MAP was defined as the sum of obtained MAPs divided by their obtained numbers of times during 6, 12, 24, 48, 72, and 96 h after cardiac arrest, respectively. The initial Sequential Organ Failure Assessment score was measured using the worst value from each scoring item within 24 h after ECPR [10]. The vasoactive inotropic score was calculated as: dopamine dose (μg/kg/min) + dobutamine dose (μg/kg/min) + 100 × epinephrine dose (μg/kg/min) + 10 × milrinone dose (μg/kg/min) + 10,000 × vasopressin dose (unit/kg/min) + 100 × norepinephrine dose (μg/kg/min) [11,12].

The primary outcome was neurological status upon discharge, as assessed by the Glasgow-Pittsburgh Cerebral Performance Categories (CPC) scale (range from 1 to 5) [13]. CPC scores of 1 and 2 were classified as favorable neurologic outcomes and CPC scores of 3 to 5 as poor neurologic outcomes. We thoroughly reviewed the medical records of the patients and they were graded based on the CPC scale by two independent intensivists (JAR and YIL).

### 2.3. Statistical Analyses

All data are presented as medians and interquartile ranges (Q1~Q3) for continuous variables and as numbers (percentages) for categorical variables. Data were compared using the Mann-Whitney *U* test for continuous variables and the chi-squared test or Fisher’s exact test for categorical variables, as appropriate. Variable importance was estimated through several machine learning methods (Bagging, Random Forest, Boosting) to predict risk factors associated with poor neurological outcomes [14,15,16]. Since the variable importance metrics appeared slightly different for each algorithm, the union of these variables became a group of candidate variables for evaluating statistical significance. Using the candidate variables selected above, an interpretable model was constructed using multiple logistic regression. In the logistic regression model, variables that did not show statistical significance or variables that slightly change area under the curve were additionally removed to estimate the final model. Spline curves were drawn to evaluate graphically the effect of average MAPs measured at several specific time points on poor neurologic outcomes [17]. Cumulative incidences of mortality were calculated by Kaplan–Meier estimates and compared using a log-rank test. All tests were two-sided and *p* values of less than 0.05 were considered statistically significant. Statistical analyses were performed with R Statistical Software (version 4.0.2; R Foundation for Statistical Computing, Vienna, Austria) and GraphPad Prism 8 (GraphPad Software, San Diego, CA, USA).

## 3. Results

### 3.1. Baseline Characteristics and Clinical Outcomes

Finally, 253 patients were analyzed in this study (Figure 1). The median age of the patients was 61 (51–71) years and 182 patients (71.9%) were men. Although 20 patients (7.9%) had a history of stroke, they were able to perform activities of daily living independently before cardiac arrest. Forty-seven patients (18.6%) experienced out-of-hospital cardiac arrest. The median CPR duration was 23.0 (11.0–36.0) min. The baseline characteristics of the ECPR patients are presented in Table 1. Compared with the favorable neurologic outcome group, the poor neurologic outcome group had older patients, higher incidence of out-of-hospital cardiac arrest, longer CPR duration, and higher Sequential Organ Failure Assessment score.

Among 253 patients, 144 (56.9%) survived until discharge from the hospital. Of those, 104 patients had favorable neurologic outcomes. Forty patients with CPC scale 3 (*n* = 7) or 4 (*n* = 33) survived till discharge though they had poor neurologic outcomes. The entire distribution of CPC scales is shown in Figure 1.

### 3.2. The Relationship between Mean Arterial Pressure and Neurologic Outcomes

**Figure 2:**
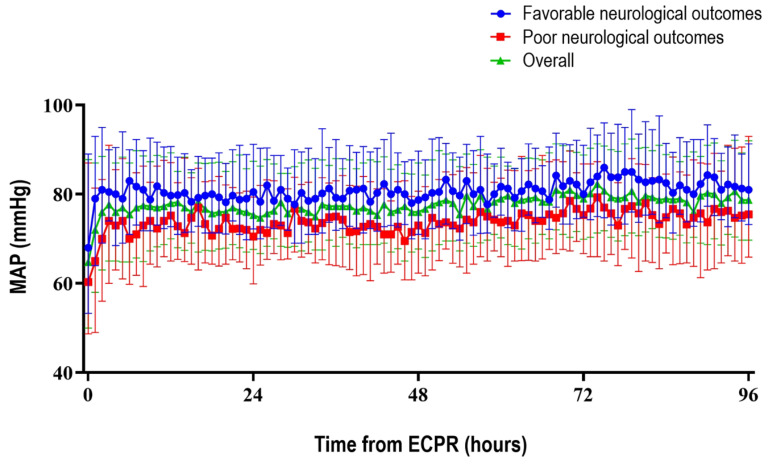
The trends of mean arterial pressure (MAP) after extracorporeal cardiopulmonary resuscitation (ECPR).

Figure 2 shows hourly average MAP based on the neurological outcomes during the first 96 h for ECPR patients. Overall, patients with favorable neurological outcomes had higher average MAP than those with poor neurological outcomes. Age, GCS on ICU admission, CPR duration, and average MAPs were identified as important variables in ensemble algorithms for machine learning (Figure S1). The value of average MAP demonstrated changes in accordance with the observation time. Overall, six models were established with observation times of 6, 12, 24, 48, 72, and 96 h from ECPR (Table 2).

In multivariable analysis, CPR duration, GCS, and average MAPs were significant risk factors of poor neurologic outcomes in all the models (all *p* < 0.05). Except in the case of models 5 and 6 (*p* = 0.055 and *p* = 0.078, respectively), old age was also identified to be significantly associated with poor neurological outcomes. The performance of model 6 (area under curve = 0.878, Akaike information criteria = 223.3, χ^2^ = 0.406) was best to predict poor neurological outcomes among the six models. However, the predictive performance of all the models was high (area under curves of all models > 0.85), and there was no significant difference in the predictive performance between each model (Table 2).

**Figure 3:**
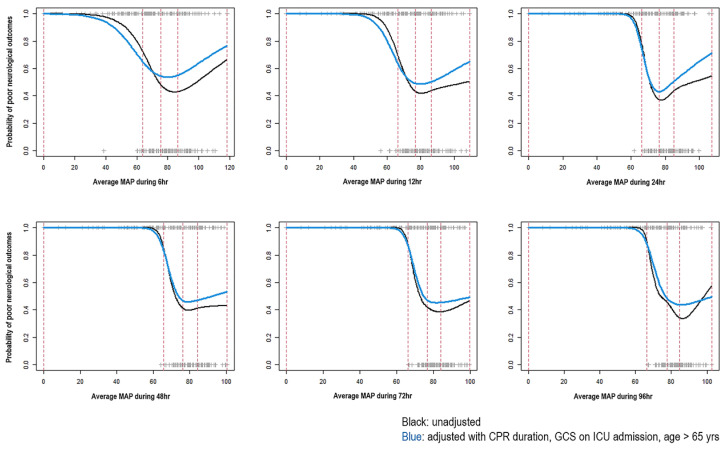
Spline curves of average mean arterial pressure (MAP) and poor neurologic outcomes according to the observation time. CPR, cardiopulmonary resuscitation; GCS, Glasgow Coma Scale; ICU, intensive care unit.

The spline curves of average MAP at specific times and poor neurologic outcomes according to each model are presented in Figure 3. The patients with average MAP around 75 mmHg demonstrated the least probability of poor neurologic outcomes in all the models. However, those with average MAPs below 60 mmHg had a high probability of poor neurological outcomes. In addition, according to an increase in the average MAP, the risk of poor neurological outcomes tended to increase in patients with an average MAP greater than 75 mmHg.

**Figure 4:**
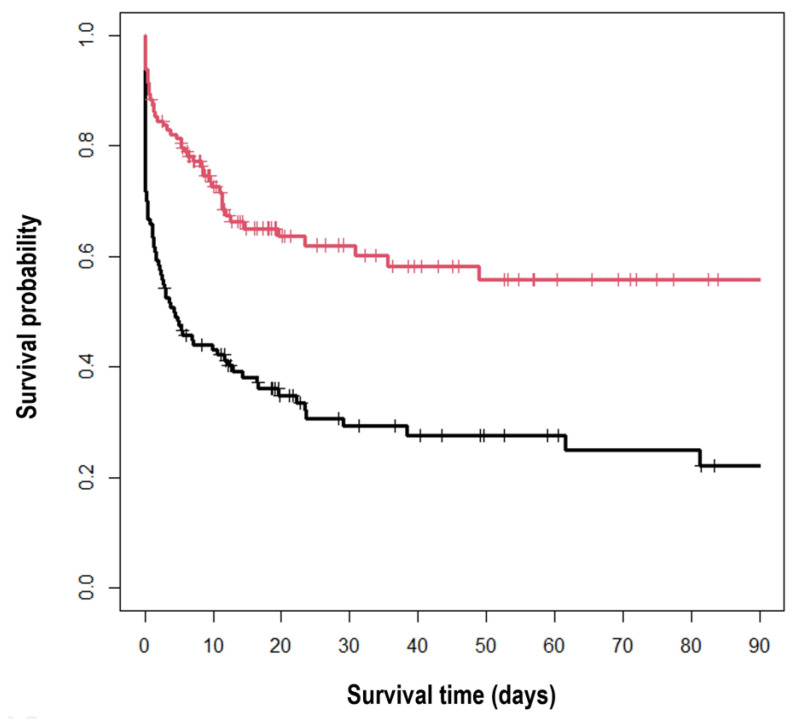
Kaplan Meier curves of 90-day mortality according to the average mean arterial pressure (MAP) during six hours. Red: average MAP ≥ 75 mmHg, black: average MAP < 75 mmHg (*p* < 0.001).

The Kaplan Meier curves of 90-day mortality revealed that patients with MAP 75 mmHg or higher during six hours had significantly better survival compared to the patients with MAP less than 75 mmHg (56% vs. 22%, log-rank test, *p* < 0.001) (Figure 4).

## 4. Discussion

In the present study, we investigated the predictors of poor neurologic outcomes and optimal MAP target for patients who underwent ECPR. The major findings were as follows: First, significant variables of the risk prediction model for poor neurological outcomes by ensemble algorithms for machine learning and multiple logistic regression included the old age, GCS on ICU admission, CPR duration, and average MAPs. Second, six models were composed by ensemble algorithms and logistic regression according to specific times. There were no significant differences in the predictive performances of poor neurological outcomes between each model. Therefore, it is proposed that observing a patient’s MAP during the first 6 h after ECPR might not be substandard to predict the ECPR patient’s neurologic outcomes than observing it during 96 h. Third, the patients with average MAP around 75 mmHg demonstrated the least probability of poor neurologic outcomes in all the models. However, those with average MAPs below 60 mmHg had a high probability of poor neurological outcomes. Based on an increase in average MAP, the risk of poor neurological outcomes tended to increase in patients with average MAP greater than 75 mmHg.

Similar to previous studies, age GCS on ICU admission and CPR duration were associated with clinical outcomes of patients who experienced ECPR in this study [1,10,18]. However, prior to the present study, there were limited data on optimal MAP and its maintenance duration for favorable neurological outcomes after ECPR.

The current guideline only recommends circumvention and immediate correction of hypotension, such as systolic blood pressure less than 90 mmHg or MAP less than 65 mmHg, during post-cardiac arrest care [6,7]. However, it is not clear whether only avoiding hypotension is the best treatment for a favorable neurological prognosis. Usually, the cerebral autoregulation curve can shift to the right in survivors with preserved autoregulation after cardiac arrest [2]. Therefore, MAP should be maintained at a higher level than generally accepted to ensure cerebral perfusion to adapt to altered cerebral autoregulation after cardiac arrest [2]. Indeed, several studies on survivors after conventional CPR reported favorable neurologic outcomes with higher blood pressure than that of recommended current guidelines [4,9,19,20,21]. In addition, MAP higher than 65 mmHg was also identified to be associated with favorable neurologic outcomes in the studies that employed neuro-monitoring devices [5,22]. A recent publication identified the optimal MAP as 89 mmHg in post-cardiac arrest survivors using near-infrared spectroscopy [5]. Another pilot study using brain tissue regional saturation of oxygen revealed the mean optimal MAP to be 76 mmHg [22]. In the present study, the ECPR survivors with an average MAP of 75 mmHg demonstrated the least probability of poor neurologic outcomes.

However, the association between extremely high MAP and favorable neurological outcomes in survivors after ECPR remains unclear. In this study, some patients with extremely high MAPs over 100 mmHg had poor neurological outcomes. We hypothesized two reasons for the poor outcomes in survivors with high MAPs. First, high MAPs may be associated with significant bleeding complications such as intracerebral hemorrhage in survivors with anticoagulation or coagulation abnormality due to the use of ECMO. Second, when cerebral autoregulation is impaired, high MAPs may increase CBF and intracranial pressure. Therefore, high MAPs may exacerbate cerebral edema in survivors after ECPR [23]. However, in this study, routine brain imaging or brain perfusion scans could not be performed in all the survivors with MAPs over 100 mmHg due to the risk of complications from intra-hospital transport during ECMO support.

The results showed that according to an increase in the average MAP, the risk of poor neurological outcomes tended to increase in patients with an average MAP over 75 mmHg. During the early short duration of MAP measurements, the risk of poor neurological outcomes tended to increase when the average MAP was greater than 75 mmHg. However, this tendency was not obvious in the long duration of MAP measurement. Since a small number of the patients had high average MAP after ECPR, the spline curve could respond sensitively to a few events. The patterns of covariate-adjusted curves were also similar to those of spline curves. Although the risk of poor neurological outcomes tended to increase in patients with average MAPs over 75 mmHg, it was not obvious whether average MAP over 75 mmHg was statistically associated with poor neurological outcomes in this study.

To prevent secondary cerebral injury, which is the additive cerebral injury characterized by an imbalance between post-resuscitation cerebral oxygen delivery and use, proper oxygen delivery with optimal CBF is important in survivors after ECPR [1]. At an early stage, maintenance of proper blood pressure is important to secure CBF and to prevent secondary cerebral injury in the patients. Previous publications revealed the association between neurologic outcomes and MAP during the first 6 h from CPR [4,9,21,24]. In this study, average MAP during the initial 6 h was also identified to be associated with neurological outcomes in patients after ECPR. In addition, it is hypothesized that the predictive value of average MAP during the initial 6 h might not be substandard to those of 12, 24, 48, 72, and 96 h. Therefore, secondary brain injury at an early stage may be significantly associated with neurological outcomes in survivors after ECPR.

This study has several limitations. First, this was a retrospective review; thus, the CPC score was determined based on medical records. Second, this study was conducted over a long time at a single institution. During that time, post-cardiac arrest care might have been more advanced than in the past, which might have affected patient’s outcomes during the study period. Third, the effects of continuous ECMO flow on cerebral autoregulation were unknown in this study. Lastly, this study lacks a tool on an external validation cohort to overcome the possible limitations of the external validation of our model.

## 5. Conclusions

In this study, it is hypothesized that average MAP during the initial 6 h after ECPR may be associated with secondary cerebral injury. Further, the patients’ outcomes could be improved by the effort to maintain MAP over 75 mmHg. Though old age, GCS on ICU admission, and CPR duration were identified to be associated with primary cerebral injury or neurological outcomes, these variables could not be improved after return of spontaneous circulation. Therefore, maintaining the survivor’s MAP at about 75 mmHg may be important for neurological recovery after ECPR.

## Figures and Tables

**Table 1:** Baseline Characteristics of the patients.

	Favorable Neurologic Outcomes (*n* = 104)	Poor Neurologic Outcomes (*n* = 149)	*p*-Value
Age (years)	60.0 (49.8–70.3)	63.0 (52.0–72.0)	0.061
Old age (age > 65 years)	33 (31.7)	68 (45.6)	0.028
Sex, male	79 (76.0)	103 (69.1)	0.295
Body surface area (m^2^)	1.8 (1.6–1.9)	1.8 (1.6–1.9)	0.898
Medical history			
Diabetes mellitus	31 (29.8)	50 (33.6)	0.623
Hypertension	46 (44.2)	75 (50.3)	0.407
Malignancy	13 (12.5)	32 (21.5)	0.095
Dyslipidemia	17 (16.3)	18 (12.1)	0.434
Current smoker	27 (26.0)	24 (16.1)	0.078
Chronic kidney disease ^a^	9 (8.7)	17 (11.4)	0.617
Previous myocardial infarction	19 (18.3)	35 (23.5)	0.400
Previous stroke	10 (9.6)	10 (6.7)	0.545
CPR details			
Out-of-hospital cardiac arrest	10 (9.6)	37 (24.8)	0.004
Home	4 (3.8)	18 (12.0)	
Public places	5 (4.8)	9 (6.0)	
Workplaces	1 (1.0)	7 (4.7)	
Others	0 (0)	3 (2.0)	
In-hospital cardiac arrest	94 (90.4)	112 (75.2)	0.004
ICU	32 (30.8)	67 (45.0)	
Emergency department	26 (25)	13 (8.7)	
Cardiac catheterization lab	34 (32.7)	11 (7.4)	
Others (operation room, general wards etc.)	2 (1.9)	21 (14.1)	
Bystander performed CPR	103 (99.0)	137 (91.9)	0.026
Initial shockable rhythm,	40 (38.5)	40 (26.8)	0.069
CPR duration (min)	12.5 (5.0–22.3)	31.0 (20.0–43.0)	<0.001
Targeted temperature management	21 (20.2)	28 (18.8)	0.908
Glasgow Coma Scale	3.0 (3.0–9.0)	3.0 (3.0–3.0)	<0.001
SOFA score	12.0 (11.0–14.0)	14.0 (12.0–15.0)	<0.001
Management in the intensive care unit			
Continuous renal replacement therapy	30 (28.8)	67 (45.0)	0.014
Vasopressor	98 (94.2)	143 (96.0)	0.733
Intra-aortic balloon counterpulsation	5 (4.8)	5 (3.4)	0.798
Mechanical ventilator	82 (78.8)	120 (80.5)	0.865
ECMO duration (h)	52.6 (22.8–105.3)	44.7 (8.7–102.6)	0.207
Maximal ECMO flow index during 6 h ^b^ (L/min/m^2^)	1.9 (1.6–2.2)	1.8 (1.1–2.1)	0.036
Maximal vasoactive score during 6 h	10.0 (0.0–31.8)	20.0 (0.0–65.0)	0.003
ECMO complications			
Limb ischemia	4 (3.8)	12 (8.1)	0.276
ECMO site bleeding	13 (12.5)	18 (12.1)	0.276
Stroke	5 (4.8)	9 (6.0)	0.887
Gastrointestinal bleeding	1 (1.0)	9 (6.0)	0.087
Sepsis	0 (0.0)	6 (4.0)	0.099
Average MAP			
During 6 h	80.5 (72.8–91.0)	69.8 (56.7–82.4)	<0.001
During 12 h	81.6 (74.1–88.0)	70.7 (57.2–84.4)	<0.001
During 24 h	80.7 (75.6–87.2)	68.7 (58.3–82.6)	<0.001
During 48 h	80.4 (75.7–86.4)	70.1 (57.7–81.0)	<0.001
During 72 h	81.5 (76.1–86.2)	69.2 (56.9–80.4)	<0.001
During 96 h	82.6 (76.5–86.3)	69.5 (56.9–79.7)	<0.001

^a^ Chronic kidney disease is defined as either kidney damage or GFR <60 mL/min/1.73 m^2^ for ≥3 months. ^b^ Maximal ECMO flow index is defined as maximal ECMO flow (l/min) divided in body surface area (m^2^). Reported are n (%) for categorical variables and median (Q1~Q3) for continuous variables. CPR, cardiopulmonary resuscitation; SOFA, Sequential Organ Failure Assessment; ECMO, extracorporeal membrane oxygenation; MAP, mean arterial pressure.

**Table 2:** Multiple logistic regression models according to the observation time.

Model	Observation Time	Variables	Odds Ratio (95% CI)	*p*-Value	R-Square	AIC ^b^	AUC
Model 1	During 6 h	CPR duration	1.081 (1.054–1.110)	<0.001	0.362	237.9	0.859
		GCS on ICU admission	0.807 (0.725–0.899)	<0.001			
		Old age ^a^	2.344 (1.175–4.675)	0.016			
		Average MAP during 6 h	0.980 (0.962–0.998)	0.031			
Model 2	During 12 h	CPR duration	1.074 (1.048–1.101)	<0.001	0.351	241.6	0.853
		GCS on ICU admission	0.825 (0.742–0.918)	<0.001			
		Old age ^a^	2.198 (1.122–4.308)	0.022			
		Average MAP during 12 h	0.970 (0.948–0.993)	0.010			
Model 3	During 24 h	CPR duration	1.073 (1.047–1.100)	<0.001	0.383	231.7	0.867
		GCS on ICU admission	0.821 (0.739–0.913)	<0.001			
		Old age ^a^	2.040 (1.034–4.025)	0.040			
		Average MAP during 24 h	0.958 (0.932–0.984)	0.002			
Model 4	During 48 h	CPR duration	1.072 (1.045–1.099)	<0.001	0.387	229.5	0.867
		GCS on ICU admission	0.830 (0.747–0.923)	<0.001			
		Old age ^a^	2.005 (1.007–3.990)	0.048			
		Average MAP during 48 h	0.939 (0.910–0.970)	<0.001			
Model 5	During 72 h	CPR duration	1.072 (1.045–1.099)	<0.001	0.399	225.5	0.874
		GCS on ICU admission	0.832 (0.748–0.925)	<0.001			
		Old age ^a^	1.972 (0.985–3.947)	0.055			
		Average MAP during 72 h	0.930 (0.899–0.963)	<0.001			
Model 6	During 96 h	CPR duration	1.072 (1.045–1.100)	<0.001	0.406	223.3	0.878
		GCS on ICU admission	0.833 (0.749–0.927)	<0.001			
		Old age ^a^	1.875 (0.932–3.770)	0.078			
		Average MAP during 96 h	0.926 (0.894–0.959)	<0.001			

^a^ Old age is defined as age > 65 years. ^b^ The smaller the AIC, the better the goodness of fit. CI, Confidence interval; AIC, Akaike information criteria; AUC, Area under the curve; CPR, Cardiopulmonary resuscitation; GCS, Glasgow Coma Scale; MAP, Mean arterial pressure.

## Data Availability

Regarding data availability, our data are available on the Harvard Dataverse Network (http://dx.doi.org/10.7910/DVN/TUDD2K, accessed on 28 April 2021).

## Supplementary Materials

The following supporting information can be downloaded at: https://www.mdpi.com/article/10.3390/jcm11020290/s1, Figure S1: Variable importance by machine learning methods. It was estimated through Bagging (A), Random Forest (B) and Boosting (C) to predict risk factors associated with poor neurological outcomes.

## Abbreviations

CBF, cerebral blood flow; CPC, cerebral performance categories; CPR, cardiopulmonary resuscitation; ECMO, extracorporeal membrane oxygenation; ECPR, extracorporeal cardiopulmonary resuscitation; GCS, Glasgow Coma Scale; ICU, intensive care unit; MAP, mean arterial pressure.

## References

[1] Ryu J.A., Chung C.R., Cho Y.H., Sung K., Jeon K., Suh G.Y., Park T.K., Lee J.M., Song Y.B., Hahn J.Y. (2019). Neurologic Outcomes in Patients Who Undergo Extracorporeal Cardiopulmonary Resuscitation. Ann. Thorac. Surg..

[2] Sundgreen C., Larsen F.S., Herzog T.M., Knudsen G.M., Boesgaard S., Aldershvile J. (2001). Autoregulation of cerebral blood flow in patients resuscitated from cardiac arrest. Stroke.

[3] Nolan J.P., Soar J., Cariou A., Cronberg T., Moulaert V.R., Deakin C.D., Bottiger B.W., Friberg H., Sunde K., Sandroni C. (2015). European Resuscitation Council and European Society of Intensive Care Medicine Guidelines for Post-resuscitation Care 2015: Section 5 of the European Resuscitation Council Guidelines for Resuscitation 2015. Resuscitation.

[4] Roberts B.W., Kilgannon J.H., Hunter B.R., Puskarich M.A., Shea L., Donnino M., Jones C., Fuller B.M., Kline J.A., Jones A.E. (2019). Association Between Elevated Mean Arterial Blood Pressure and Neurologic Outcome After Resuscitation From Cardiac Arrest: Results From a Multicenter Prospective Cohort Study. Crit. Care Med..

[5] Sekhon M.S., Gooderham P., Menon D.K., Brasher P.M.A., Foster D., Cardim D., Czosnyka M., Smielewski P., Gupta A.K., Ainslie P.N. (2019). The Burden of Brain Hypoxia and Optimal Mean Arterial Pressure in Patients With Hypoxic Ischemic Brain Injury After Cardiac Arrest. Crit. Care Med..

[6] Callaway C.W., Donnino M.W., Fink E.L., Geocadin R.G., Golan E., Kern K.B., Leary M., Meurer W.J., Peberdy M.A., Thompson T.M. (2015). Part 8: Post-Cardiac Arrest Care: 2015 American Heart Association Guidelines Update for Cardiopulmonary Resuscitation and Emergency Cardiovascular Care. Circulation.

[7] Panchal A.R., Bartos J.A., Cabanas J.G., Donnino M.W., Drennan I.R., Hirsch K.G., Kudenchuk P.J., Kurz M.C., Lavonas E.J., Morley P.T. (2020). Part 3: Adult Basic and Advanced Life Support: 2020 American Heart Association Guidelines for Cardiopulmonary Resuscitation and Emergency Cardiovascular Care. Circulation.

[8] Bhate T.D., McDonald B., Sekhon M.S., Griesdale D.E. (2015). Association between blood pressure and outcomes in patients after cardiac arrest: A systematic review. Resuscitation.

[9] Kilgannon J.H., Roberts B.W., Jones A.E., Mittal N., Cohen E., Mitchell J., Chansky M.E., Trzeciak S. (2014). Arterial blood pressure and neurologic outcome after resuscitation from cardiac arrest. Crit. Care Med..

[10] Park S.B., Yang J.H., Park T.K., Cho Y.H., Sung K., Chung C.R., Park C.M., Jeon K., Song Y.B., Hahn J.Y. (2014). Developing a risk prediction model for survival to discharge in cardiac arrest patients who undergo extracorporeal membrane oxygenation. Int. J. Cardiol..

[11] Gaies M.G., Jeffries H.E., Niebler R.A., Pasquali S.K., Donohue J.E., Yu S., Gall C., Rice T.B., Thiagarajan R.R. (2014). Vasoactive-inotropic score is associated with outcome after infant cardiac surgery: An analysis from the Pediatric Cardiac Critical Care Consortium and Virtual PICU System Registries. Pediatr. Crit. Care Med..

[12] Na S.J., Chung C.R., Cho Y.H., Jeon K., Suh G.Y., Ahn J.H., Carriere K.C., Park T.K., Lee G.Y., Lee J.M. (2019). Vasoactive Inotropic Score as a Predictor of Mortality in Adult Patients With Cardiogenic Shock: Medical Therapy Versus ECMO. Rev. Esp. Cardiol. (Engl. Ed.).

[13] Cummins R.O., Chamberlain D.A., Abramson N.S., Allen M., Baskett P.J., Becker L., Bossaert L., Delooz H.H., Dick W.F., Eisenberg M.S. (1991). Recommended guidelines for uniform reporting of data from out-of-hospital cardiac arrest: The Utstein Style. A statement for health professionals from a task force of the American Heart Association, the European Resuscitation Council, the Heart and Stroke Foundation of Canada, and the Australian Resuscitation Council. Circulation.

[14] Breiman L. (1996). Bagging predictors. Mach. Learn..

[15] Breiman L. (2001). Random Forests. Mach. Learn..

[16] Dietterich T.G. (2000). An Experimental Comparison of Three Methods for Constructing Ensembles of Decision Trees: Bagging, Boosting, and Randomization. Mach. Learn..

[17] Perperoglou A., Sauerbrei W., Abrahamowicz M., Schmid M. (2019). A review of spline function procedures in R. BMC Med. Res. Methodol..

[18] Hifumi T., Kuroda Y., Kawakita K., Sawano H., Tahara Y., Hase M., Nishioka K., Shirai S., Hazui H., Arimoto H. (2015). Effect of admission Glasgow coma scale motor score on neurological outcome in out-of-hospital cardiac arrest patients receiving therapeutic hypothermia. Circ. J..

[19] Russo J.J., James T.E., Hibbert B., Yousef A., Osborne C., Wells G.A., Froeschl M.P., So D.Y., Chong A.Y., Labinaz M. (2017). Impact of mean arterial pressure on clinical outcomes in comatose survivors of out-of-hospital cardiac arrest: Insights from the University of Ottawa Heart Institute Regional Cardiac Arrest Registry (CAPITAL-CARe). Resuscitation.

[20] Kaji A.H., Hanif A.M., Thomas J.L., Niemann J.T. (2011). Out-of-hospital cardiac arrest: Early in-hospital hypotension versus out-of-hospital factors in predicting in-hospital mortality among those surviving to hospital admission. Resuscitation.

[21] Annoni F., Dell’Anna A.M., Franchi F., Creteur J., Scolletta S., Vincent J.L., Taccone F.S. (2018). The impact of diastolic blood pressure values on the neurological outcome of cardiac arrest patients. Resuscitation.

[22] Sekhon M.S., Smielewski P., Bhate T.D., Brasher P.M., Foster D., Menon D.K., Gupta A.K., Czosnyka M., Henderson W.R., Gin K. (2016). Using the relationship between brain tissue regional saturation of oxygen and mean arterial pressure to determine the optimal mean arterial pressure in patients following cardiac arrest: A pilot proof-of-concept study. Resuscitation.

[23] Rangel-Castilla L., Gopinath S., Robertson C.S. (2008). Management of intracranial hypertension. Neurol. Clin..

[24] Laurikkala J., Wilkman E., Pettila V., Kurola J., Reinikainen M., Hoppu S., Ala-Kokko T., Tallgren M., Tiainen M., Vaahersalo J. (2016). Mean arterial pressure and vasopressor load after out-of-hospital cardiac arrest: Associations with one-year neurologic outcome. Resuscitation.

