# Supplementary material for: Optimal Mean Arterial Pressure for Favorable Neurological Outcomes in Survivors after Extracorporeal Cardiopulmonary Resuscitation
Source: J Clin Med. 2022 Jan 6;11(2):290. doi: 10.3390/jcm11020290 (PMC8779237; doi:10.3390/jcm11020290)

Supplementary Material

Figure S1. Variable importance by machine learning methods. It was estimated through Bagging (A), Random Forest (B) and Boosting (C) to predict risk factors associated with poor neurological outcomes. CPR, cardiopulmonary resuscitation; MAP, mean arterial pressure; SOFA, Sequential Organ Failure Assessment; VIS, vasoactive inotropic score; ECMO, extracorporeal cardiopulmonary resuscitation.

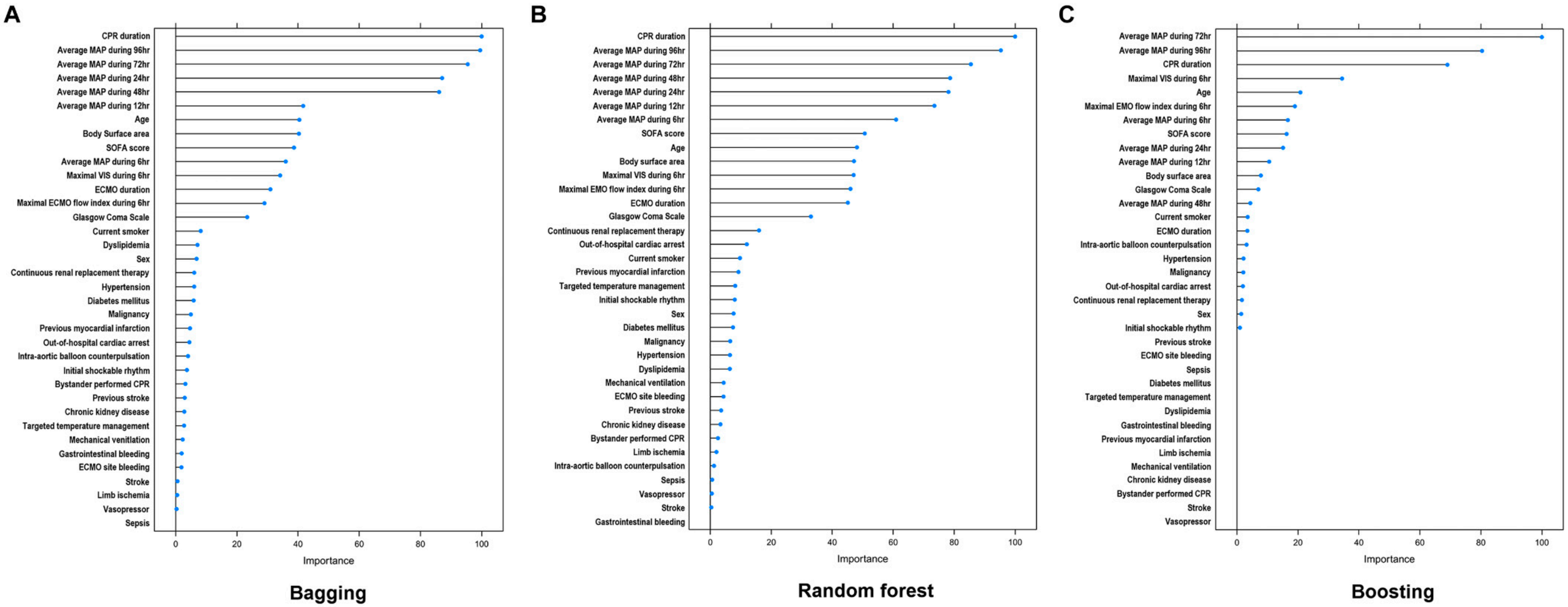

Supplement: Supplementary file 1 [file jcm-11-00290-s001.zip › jcm-1537052-supplementary.pdf]
